# The Effect of Combined Oral Contraceptive Pills on Beclin-1 and LC3B Transcript Levels in Ovarian Endometrioma

**DOI:** 10.1155/2021/5519538

**Published:** 2021-06-28

**Authors:** Wanwisa Waiyaput, Ongarj Bovornsakulvong, Srithean Lertvikool, Areepan Sophonsritsuk

**Affiliations:** ^1^Office of Research Academic and Innovation, Faculty of Medicine, Ramathibodi Hospital, Mahidol University, Bangkok 10400, Thailand; ^2^Reproductive Endocrinology and Infertility Unit, Department of Obstetrics and Gynaecology, Faculty of Medicine Ramathibodi Hospital, Mahidol University, Bangkok 10400, Thailand

## Abstract

**Background:**

Autophagy is likely altered in patients with endometriosis. Ovarian steroid hormones seem to affect this changing of the autophagic process.

**Objective:**

To study the effect of combined oral contraceptive (COC) pills on the expression of autophagic-related gene *BECN1* and *LC3B* in the ectopic and eutopic endometria of patients with endometriosis. *Material and Methods*. The present quasiexperimental study recruited 36 women (18–45 years old) with endometrioma and nonendometrioma who were scheduled for surgery. Patients with endometrioma were randomly assigned to either a no-treatment group (*n* = 12) or a COC group (*n* = 12). The COC group was prescribed a daily oral pill composed of 3 mg drospirenone and 0.03 mg ethinyl estradiol for 6 weeks before surgery. The control group (*n* = 12) was composed of women without endometrioma. Ectopic endometriotic and endometrium tissues were collected from the no-treatment and COC groups, whereas the only endometrium was collected from the control group. These tissues were used for real-time PCR to measure the expression of the *BECN1* and *LC3B* genes.

**Results:**

The baseline demographic data were not different among the three groups. The *BECN1* gene expression in endometrium tissue in the COC group was significantly less than that in the no-treatment and control groups (*P* = 0.011 and 0.029, respectively). No significant difference of endometriotic cyst *BECN1* and *LC3B* gene expression was found between COC and no treatment.

**Conclusions:**

Oral COC pills for 6 weeks continuously before surgery decreased the eutopic endometrial expression (mRNA) of the *BECN1* gene compared to those from healthy normal women and nontreated patients with an endometriotic cyst. The change in the expression of autophagy-related genes was more distinct in eutopic than ectopic endometria. This trial is registered with TCTR20170720002. Registered and enrolled the first patient on 20 July 2017.

## 1. Introduction

Endometriosis is a common gynecologic disease. Women are diagnosed with endometriosis when the histological presence of the endometrial glands and stroma tissue outside of the uterine cavity, such as implant of the ectopic endometrial in pelvic peritoneum, fallopian tubes, and ovaries, is identified. Several theories have been proposed for the pathophysiology of the disease, for example, retrograde menstruation, embryonic rests, and induction theory. However, no single theory can account for the various locations of endometriosis existing in many patients [1, 2].

One of the important emerging mechanisms to control the production and destruction of many cells in the body is known as the “self-eating process” or “autophagy.” Autophagy is the cytoprotective process of the cells, degrading damaged cells and redundant organelles, and recycling cellular organelles. Autophagy also plays a critical role in regulating physiologic processes, such as tumorigenesis, embryo development, and tissue remodeling in many systems, including the female reproductive tract [3]. The response of autophagy is influenced by hormone and cellular stress from nutrient deprivation to maintain cellular homeostasis. The initial process of autophagy occurs following the activation of uncoordinated- (Unc-) 51-like autophagy-activating kinase (ULK) 1/2 protein assisted by protein Beclin-1, yeast vacuolar *protein* sorting (Vps) 34, and autophagy-related protein (Atg) 9, which leads to the complex formation of a phagophore from the cellular endoplasmic reticulum, mitochondria, or plasma membrane. Human Beclin-1 is a protein encoded by the *BECN1* gene. The elongation of phagophore is regulated by two protein complexes: Atg12-Atg5-Atg16 and phosphatidylethanolamine- (PE-) conjugated-microtubule-associated protein 1A/1B-light chain 3- (LC3-) II (or Atg8). LC3 (Autophagy Marker Light Chain 3B, MAP1A/MAP1B LC3 B) in humans is encoded by the gene *MAP1LC3B* or *LC3B* (microtubule-associated proteins 1A/1B light chain 3B). LC3-II originates from the conjugation of LC3-I to PE [3, 4]. The isolated membrane then surrounds the cargo to form the autophagosome. The LC3-II is then cleaved from the outer membrane of the autophagosome. The fusion of the autophagosome with the lysosome, the so-called autolysosome, leads to the degradation of the cargo by the lysosomal hydrolase enzyme [4].

The current recent evidence from many studies demonstrated that autophagy in the human endometrium occurs in intracycle variation associated with the phase of the menstrual cycle to maintain homeostasis of the endometrium. The study showed that the autophagy process increases significantly in the secretory menstrual phase of menstruation much more than in the proliferative phase. These changes are associated with the process of apoptosis on the endometrial cells in a cyclic pattern [5]. This process is likely altered in patients with endometriosis. Recent studies have reported that endometrial *BECN1* is downregulated in the patient diagnosed with adenomyosis [6] and endometrioma [7] compared with normal endometrium from women without endometrioma.

The differential role of estrogen and progesterone on autophagy in endometrial and endometriotic cells has been demonstrated. An *in vitro* study using Ishikawa endometrial cells demonstrated that autophagy was induced when progesterone was added to the estrogen-treated endometrial cells (the proliferative phase) and maximally increased when estrogen and/or progesterone was removed (the menstrual phase) from the estrogen- and progesterone-treated endometrial cells (the secretory phase) [5]. However, a different type of progesterone exerted a different effect on autophagy in endometriotic cells *in vitro* [8]. Natural progesterone, as well as estrogen alone, cannot induce the autophagy process, which is different from synthetic progestin (dienogest).

We performed an extensive literature search, and no study has ever been published regarding the effects of combined hormonal contraception on autophagy in patients with ovarian endometrioma before surgery. Therefore, here, we study the effect of combined oral contraceptive (COC) pills on autophagy-related genes in patients with ovarian endometrioma. *BECN1* and *LC3B* expressions are widely used as autophagic markers [9]. The enhancement of Beclin-1 and LC3 expression is necessary for autophagy, while the downregulation of Beclin-1 also induces the apoptosis process [10]. LC3 was upregulated during autophagy induction [3, 11].

## 2. Material and Methods

The study was approved by the Ethical Clearance Committee on Human Rights Related to Research Involving Human Subjects, Faculty of Medicine Ramathibodi Hospital. This study followed the recommendations of the Consolidated Standards of Reporting Trials (CONSORT-statement). All 36 eligible women (18–45 years old) consented to participate in the study.

Women who had at least one ovarian cyst, either unilateral or bilateral compatible with endometriosis diagnosed by ultrasonography (*n* = 24), were recruited into endometriosis groups, while women who had no ovarian endometriotic cyst but required other benign adnexa surgery or tubal reanastomosis were recruited into the control group (control group, *n* = 12). Participants with ovarian endometriotic cysts were further randomly allocated preoperatively into two groups, receiving either oral COC pills containing 0.03 mg ethinyl estradiol and 3 mg drospirenone per day 6 weeks before surgery (COC group, *n* = 12) or nothing (no-treatment group, *n* = 12). The participants in the COC group started taking medication within the first 5 days of their menstruation. Serial numbered opaque and sealed envelopes were established according to a computer-generated block of four randomizations. The envelopes were opened by a physician at the outpatient department unit after enrollment. These participants underwent laparoscopic or laparotomy surgery from August 2017 to March 2018 at the Department of Obstetrics and Gynaecology, Faculty of Medicine Ramathibodi Hospital. The surgery was scheduled during the early to the midfollicular phase of the menstrual cycle for the participants in no treatment and control groups or 6 weeks after continuous COC use for the participants from the COC group. The subjects were excluded if the final histopathological report of the ovarian cyst was not endometriosis.

After allocation, all women had anthropometric measurements taken, including body weight and height. Each participant was questioned about preoperative symptoms as the baseline characteristic assessment. Endometrium tissue approximately 0.5 × 0.5 × 0.5 cm^3^ was collected with Sim uterine curettage No. 0 after anesthesia, and endometriotic cyst wall size 2.5 × 2.5 cm^2^ was collected after cystectomy without using electrocautery. Tissues were then placed in RNA*later*® solution (Ambion, Austin, TX, USA) for 24 h at 4°C before storing frozen at −80°C until analysis. Total RNA was extracted from endometriotic tissues by the RNeasy Fibrous Tissue Mini Kit (Qiagen, Hilden, Germany). The RNA concentration and quality were determined by measuring the absorbance at 260 and 280 nm. Total RNA (1 *μ*g) was reversed-transcribed to generate a cDNA library using an ImProm-II™ Reverse Transcription System (Promega, Madison, Wisconsin, USA). Real-time reverse transcriptase-polymerase chain reaction (PCR) for Beclin-1 and LC3 transcripts was performed with the CFX96 Real-Time PCR Instrument (Bio-Rad Laboratories, Inc., Hercules, CA, USA) using the SoFastTM EvaGreen Supermix (Bio-Rad Laboratories, Inc., Hercules, CA, USA) and primers (Integrated DNA Technologies, Inc., Coralville, USA) [12]. The PCR reactions were performed for 35 cycles at 95°C for 3 min, 59°C for 5 s using primers specific for *BECN1*, *LC3B*, *β-actin*, and glyceraldehyde-3-phosphate dehydrogenase (*GAPDH*). The primers used for amplification were as follows: *BECN1*: forward, 5′-TAG ACC GGA CTT GGG TGA CG-3′, reverse, 5′-TAG ACC CTT CCA TCC CTC AGC-3′; *LC3B*: forward, 5′-CCG CAC CTT CGA ACA AAG AG-3′, reverse, 5′-AAG CTG CTT CTC ACC CTT GT-3′ [13]; *β-actin*: forward, 5′-TCCTTCCTGGGCATGGAG-3′, reverse, 5′-GATGTCCACGTCACACTTCA-3′; and *GAPDH*: forward, 5′-GAA GGT GAA GGT CGG AGT C-3′, reverse, 5′-GAA GAT GGT GAT GGG ATT TC-3′ [13]. The real-time PCR results for *BECN1* and *LC3B* were normalized with geometric means of *β-actin*, and *GAPDH* gene expression and data were present as relative gene expression.

### 2.1. Statistical Analysis

Statistical analysis was performed using IBM SPSS Statistics for Windows, Version 22.0 (IBM Corp, Armonk, NY, USA). A Shapiro-Wink test was applied to a normality test. ANOVA was used for the comparison of continuous variables in parametric data, and the Kruskal-Wallis test has used the comparison of continuous variables in nonparametric data. Data were presented as means ± standard deviation (SD) and number (%). ANOVA and multiple comparisons were used to investigate the expression of *BECN1* and *LC3B* gene expressions between each group. The results were considered statistically significant at *P* < 0.05.

## 3. Results

**Figure 1:**
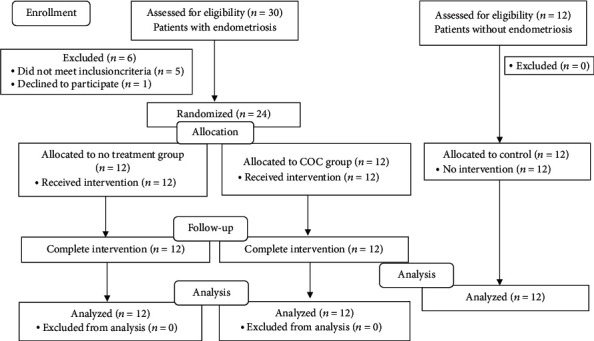
The study flow chart.

Overall, 24 patients with endometriotic cyst underwent randomization. The 12 patients without endometriotic cyst were included in the control group. The flow of participants is summarized in Figure 1. The characteristics of the patients and baseline operative indication and intraoperative parameters were not statistically significant among the three groups and are shown in Tables 1 and 2. The Beclin-1 and LC3 mRNA normalized by the geometric mean of the transcript level of *β*-actin and GAPDH are presented in Tables 3 and 4. The ANOVA analysis for the level of endometrial Beclin-1 but not LC3B was significantly different among the three groups. Multiple comparison analysis demonstrated that endometrial Beclin-1 transcript from participants in the COC group was significantly lower than those in the no-treatment group and control (*P* = 0.011 and 0.029, respectively) (Table 3). No significant difference of endometriotic cyst Beclin-1 but not LC3B transcripts was found between the COC and no-treatment groups (Table 4).

Interestingly, both *BECN1* and *LC3B* gene expressions from patients with endometriosis treated with COC were found to be significantly lower in endometrium than in endometriotic cyst; however, no significant difference in gene expression was detected when comparing endometriotic cyst and endometrium in patients with untreated endometriosis (Table 5).

## 4. Discussion

In this study, we demonstrate that endometrial *BECN1* gene expression in a patient treated with an oral daily dose of 0.03 mg ethinylestradiol and 3 mg drospirenone per day for 6 weeks before surgery was lower than that in the control and no-treatment groups. Moreover, *BECN1* and *LC3B* gene expressions in the endometrium were significantly lower than those in the endometriotic cyst in those patients treated with the oral pills. No such effect was reported in patients with endometriotic cyst with no treatment. However, COC pills did not modulate gene expression of *BECN1* and *LC3B* in endometriotic cyst tissue.

Autophagy, or the self-eating process, is also a potential pathogenesis of endometriosis [6–8, 14]. This process is composed of multiple complex mechanisms, and many genes are involved [11]. *BECN1* and *LC3B* are widely known genetic markers for autophagy and are related to the initiation step, as well as the elongation of autophagosome [11]. Autophagy is related to the pathogenesis and the progression of endometriosis [7, 14]. Autophagosome and *LC3B* expression in endometrial stromal cells (ESCs) of ectopic and eutopic endometria in patients with endometriosis decreased compared to those in the ESCs of eutopic endometrium in normal women [15]. Ruiz et al. reported a decrease in autophagy ectopic EECs and ESCs in either the proliferative or secretory phase when compared with the normal endometrium [16]. Similar to normal endometrium, the autophagy level was higher in eutopic ESCs in the secretory phase than in eutopic ESCs in the proliferative phase in patients with endometriosis [15]. However, the autophagy in ESC ectopic endometrium is at a nearly constant level throughout the menstrual cycle [15]. Interestingly, few studies have reported a significant increase in autophagy in endometriotic cyst compared with the eutopic endometrium of patients with endometriosis or healthy women [13].

Ovarian steroid hormone likely controls autophagy in endometrium and endometriotic cells with the differential effect of each hormone. The role of ovarian steroid hormone on autophagy is demonstrated in ovariectomized mice. With deprivation of ovarian hormone because of removal of the ovaries, autophagy is at the highest level, as demonstrated by LC3-II and Atg5 levels. The protein levels were then lowered when estradiol or progesterone was given to the animal [17]. As demonstrated by Choi et al., autophagy plays a role in the cyclical change of human endometrium obtained from the patient and human endometrial cell line. The autophagy is activated during the proliferative phase (estrogen alone effect) and increased further with the secretory phase (progesterone effect) and menstrual phase (estrogen and progesterone effects) [5]. This was closely correlated to apoptosis induction and the human endometrial cycle.

The effect of combined estrogen and progesterone in COC on autophagy in the present study was different from the effect of progestin dienogest plus estrogen on autophagy in Choi et al.'s study in 2015. The authors isolated human ESCs from the endometriotic ovarian cyst and treated them with estrogen-only, estrogen plus progesterone, and estrogen plus dienogest. At the same time, the expression of *LC3* was significantly upregulated when the cells were treated with dienogest plus estrogen but not with estrogen alone or estrogen plus progesterone [8]. No significant change due to estrogen plus progesterone could be explained by the progesterone resistance of endometriotic tissue. There are several factors that contributed to the different results between our study and Choi et al.'s study, including the type of experiment (*in vitro* vs. *in vivo*) and types of estrogen and progesterone. However, the study of the individual hormone effect on the ovariectomy mouse model is in line with our study. The level of LC3-II decreases after either 17*β* estradiol or progesterone was administered [17].

Our study demonstrates that *BECN1* and *LC3B* show a distinct profile of gene expressions in both eutopic and ectopic endometrium. It is thought that these proteins are regulated by different mechanisms [17]. Beclin-1 is required for vesicle nucleation during autophagosome formation and is an important merging point between autophagy and apoptosis because it interacts with the antiapoptotic and antiautophagic protein B-cell lymphoma- (Bcl-) 2. However, several pieces of evidence have shown that autophagy and apoptosis are two cross-talking mechanisms [18].

However, no effect of COC pills on autophagy in ectopic endometrium derived from endometriotic cyst wall, but it affected the expression of the autophagic gene in eutopic endometrium. This result is compatible with Choi et al. [14]. There was a constant activity of autophagy in endometriotic cyst tissue throughout the menstrual cycle and related to a decrease in apoptosis. Primary ESCs isolated from the endometriotic cyst and primary ESCs isolated from normal endometrium were cultured with (1) estrogen-only or (2) estrogen and progesterone. No difference in the expression of LC3-II, cleaved caspase 3, and a marker protein for mTOR in endometriotic ESCs was shown between estrogen or estrogen and progesterone administration or even when these hormones were removed from the culture medium, in contrast to the effect of sex steroid hormones on normal eutopic ESCs. The expression of LC3-II and cleaved caspase 3 increased significantly in on normal eutopic ESCs treated with estrogen and progesterone compared with estrogen alone. Removal of estrogen and progesterone after incubation of normal eutopic ESCs with both hormones enhanced the expression of *LC3* and cleaved *caspase 3* [14, 19].

Our study demonstrates that there was no significant difference in *LC3B* and *BECN1* expressions when comparing the eutopic or ectopic endometria of endometriosis patients in the no-treatment controls. Our result is incompatible with other studies [6, 7, 13, 14, 16]. This might be because the endometrial collection was performed during the proliferative menstrual phase in both control and endometriosis patients. Although the autophagic level was decreased in endometrial glandular epithelial cells (EECs) and ESCs in either the proliferative or secretory phase compared with the endometrium from controls, it is very slightly low in the proliferative phase and more significantly lower in the secretory phase [15, 16]. Therefore, such a subtle difference could not be detected among this small sample size of participants.

## 5. Limitation of This Study

In this study, we only measure the expression of two crucial genes involved in autophagy, although many genes are involved in several steps of autophagy (a complex molecular process), including initiation, phagophore expansion, autophagosome maturation, fusion with the lysosome, cargo degradation, and efflux. Although *LC3* is the most widely used gene marker for monitoring autophagy, specifically LC3-II for autophagosome maturation, increased LC3-II cannot be definitely concluded to be a result of increased autophagic flux. Either increased autophagy or a defect in autophagosome-lysosome fusion or degradation can result in the accumulation of autophagosome [20]. Therefore, other assays are now needed for validation of our findings, for example, Western blot analysis, transmission electron microscopy, green fluorescence protein-LC3 fluorescence microscopy, and measuring the ratio of LC3-II/LC3-I protein [9]. Moreover, this study examined only mRNA gene expression, not protein expression. Also, the study was done in humans, so we cannot manipulate and investigate the response of cell autophagy, such as adding a lysosomal inhibitor to the patients, as we could do when working with a cell line.

## 6. Conclusions

Continuous oral COC pills for 6 weeks before surgery decreased the eutopic endometrial mRNA expression of the *BECN1* gene compared to eutopic endometrium from healthy normal women and from patients with an endometriotic cyst. The changes of autophagy-related genes are more distinct in eutopic than ectopic endometrium.

## Figures and Tables

**Table 1:** Demographic data.

Characteristics	No treatment (*N* = 12)	COC (*N* = 12)	Control (*N* = 12)	*P*
Age (years)	32.1 ± 4.8	33 ± 5.1	34 ± 5.2	0.682
BMI (kg/m^2^)	21.2 ± 2.9	21 ± 4.6	22 ± 5	0.835
Parity (*n*)	0.1 ± 0.5	0	1.5 ± 0.9	<.001
Duration of menstruation (d)	3.7 ± 0.7	3.4 ± 0.6	3.6 ± 0.6	0.479
Interval of menstruation (d)	29.5 ± 1.4	29.3 ± 0.9	30 ± 1.4	0.467

Data are shown as mean ± SD. BMI: body mass index; COC: combined oral contraceptive.

**Table 2:** Clinical parameters for participants with endometriosis.

Characteristics	No treatment (*N* = 12)	COC (*N* = 12)	*P*
Indication of surgery, *n* (%)			
(i) Dysmenorrhea/pelvic pain	8 (66.7)	10 (83.3)	0.115
(ii) Persistent ovarian cyst	2 (16.7)	2 (16.7)	
(iii) Infertility	2 (16.7)	0	
Laterality of ovarian cyst, *n* (%)			
(i) Unilateral	10 (83.3)	9 (75)	1.000
(ii) Bilateral	2 (16.7)	3 (25)	
Diameter of ovarian cyst (cm) (mean ± SD)	4.7 ± 1.4	4.8 ± 1.3	0.884
Type of previous surgery, *n* (%)			
(i) Previous C/S	2 (16.8)	0	0.478
(ii) Previous C/S with TL	0	0	
Operative time (min) (mean ± SD)	117.5 ± 26.4	122.5 ± 30.9	0.674
Blood loss (ml) (mean ± SD)	57.9 ± 40.0	89.6 ± 67.0	0.174

COC: combined oral contraceptive; C/S: cesarean section; TL: tubal ligation.

**Table 3:** Beclin-1 and LC3 transcript levels in endometria.

	No treatment (*N* = 12)	COC (*N* = 12)	Control (*N* = 12)	*P* ^a^ value	*P* ^b^ value		
					No treatment vs. COC	COC vs. control	No treatment vs. control
mRNA expression of Beclin-1 × 10^−3^	2.4 ± 1.1	1.6 ± 0.3	2.3 ± 0.5	0.023	0.011	0.029	0.677
mRNA expression of LC3 × 10^−1^	1.2 ± 0.8	1.2 ± 0.5	1.3 ± 0.5	0.708	—	—	—

*P* ^a^: *P* value was calculated by ANOVA. *P*^b^: *P* value was calculated by LSD post hoc multiple comparisons test. mRNA: messenger ribonucleic acid; COC: combined oral contraceptive.

**Table 4:** Beclin-1 and LC3 transcript levels of endometriotic cysts.

	No treatment (*N* = 12)	COC (*N* = 12)	*P* value
mRNA level of Beclin-1 × 10^−3^	3.3 ± 1.1	3.2 ± 0.5	0.842
mRNA level of LC3 × 10^−1^	1.6 ± 0.4	1.7 ± 0.5	0.400

COC: combined oral contraceptive.

**Table 5:** Comparison of Beclin-1 and LC3 transcript levels between endometrium and endometriotic cyst for the endometriosis group.

	No treatment (*N* = 12)			COC (*N* = 12)		
	EM	Cyst	*P* value	EM	Cyst	*P*
mRNA level of Beclin-1 × 10^−3^	2.4 ± 1.1	3.3 ± 1.1	0.080	1.6 ± 0.3	3.2 ± 0.5	<0.000
mRNA level of LC3 × 10^−1^	1.2 ± 0.8	1.6 ± 0.4	0.113	1.2 ± 0.5	1.7 ± 0.5	0.021

EM: endometrium; mRNA: messenger ribonucleic acid; COC: combined oral contraceptive.

## Data Availability

Data is available on request.
